# Outcome of Endodontic Treatments Made by Postgraduate Students in the Dental Clinic of Bretonneau Hospital

**DOI:** 10.1155/2014/684979

**Published:** 2014-03-20

**Authors:** Virginie Touboul, Alice Germa, Jean-Jacques Lasfargues, Eric Bonte

**Affiliations:** ^1^Department of Conservative Dentistry and Endodontics, Dental Faculty, Descartes University Paris, 1 rue Maurice Arnoux, 92120 Montrouge, France; ^2^Bretonneau Hospital, 23 rue Joseph de Maistre, 75018 Paris, France; ^3^Department of Public Health, Dental Faculty, Descartes University Paris, 1 rue Maurice Arnoux, 92120 Montrouge, France; ^4^Department of Conservative Dentistry and Endodontics, Charles Foix Hospital, 12 avenue de la République, 94200 Ivry-sur-Seine, France

## Abstract

*Objective*. The objective of this retrospective study is double: (1) to assess the 1–4 years of outcome of endodontic treatment performed by postgraduate students in endodontics in the Dental Clinic of Bretonneau Hospital and (2) to examine outcome predictors. *Method*. 363 teeth in 296 patients were treated between 2007 and 2011. 183 patients (224 teeth) were lost during the followup. 113 patients were included in the study (recall: 38%), corresponding to 139 teeth of which 8 were extracted. 131 remaining teeth (36%) were examined clinically and radiographically. Apical periodontitis (AP) was absent (PAI = 1) or present (PAI ≥ 2). Outcome was classified as “healed,” “healing,” or “diseased”. *Results*. The success rate was 92%. No failure was observed among the 23 initial endodontic treatments. Among the 108 retreated teeth, 80% were “healed” and 11% were “healing.” An association was found between success rate and preoperative signs or symptoms (absent 95% versus present 83%), preoperative root filling density (inadequate 93% versus adequate 57%), but not between preoperative AP status and success. *Conclusion*. Outcomes in this retrospective study were similar to those previously reported. However, a larger sample size is needed to assess outcome predictors more precisely.

## 1. Introduction

Many studies have shown that endodontic treatments can provide a high rate of success despite the complexity of the endodontic space [1, 2]. Nevertheless, a wide range of results is still reported by systematic reviews using clinical and radiographic measures of periapical healing [3–5].

So, for a variety of reasons, endodontic failures still occur and some practitioners delegate endodontic treatments to qualified endodontists. Therefore, some patients are referred to the Graduate Endodontic Clinic at the Bretonneau Hospital (Paris, France) for both initial treatments and retreatments. One of the criteria of the students' evaluation is the outcome of the endodontic treatments. The management team of the hospital wanted to study the effectiveness of this clinic too; and a retrospective study was carried out. The purpose of this study was to (a) assess the 1–4-year outcome of endodontic treatment performed by postgraduate students and (b) to examine the outcome predictors after a minimum follow-up period of 1 year [6].

## 2. Materials and Methods

### 2.1. Population

A total of 296 patients (363 teeth) were treated in the Dental Clinic of Bretonneau Hospital between January 2007 and December 2010 by endodontic treatment (initial or retreatment) on one tooth or more. The sample population included all patients referred to us from general dental practice and other clinical units of the dental hospital except those who had a medical contraindication. The detailed medical and dental history was obtained from each patient. Patients were informed about the various treatment alternatives and the benefits and risks associated with each solution. Informed consent was signed by all patients before treatment.

The exclusion criteria were the patient with high risk of bacterial endocarditis or immunocompromised patients. All treated patients were in good general health, except one of them (Hodgkin lymphoma).

### 2.2. Treatment

The treatments were performed by 9 postgraduate students, supervised by qualified endodontists under operative microscope (Zeiss Pico WIPO). Aseptic techniques were systematically observed, with rubber-dam isolation, and if needed, reconstruction of missing walls with glass ionomer cement (Fuji IX, GC), and possibly a copper ring.

The root canal preparation was carried out in accordance with the biological and mechanical principles of Schilder [7]. Canals were cleaned and shaped using hand files and Protaper system. They were irrigated with a 3% sodium hypochlorite solution (Parcan Septodont) using Endoneedle 28 G (VMK) syringe. The working length was determined using an apex locator (Root ZX, Morita or Propex II Denstply) and the canal was prepared at a minus 0.5 mm with respect to the zero reading position length.

Pre, intra-, and postoperative X-rays were taken with a Rinn film-holder and operated with the VistaScan digital system (Dürr Dental). Sonic and/or ultrasonic inserts (Endo-Success kit on a suitable device PMAX, Satelec-Acteon) were used if needed.

For retreated teeth, silver points and fragments of broken instruments have been bypassed preferably with hand instruments and vibrated with ultrasonics tips. If a bypass has been obtained and if the instrument could not be removed, it was included in the filling material. Gutta-percha and sealer were removed using manual and ultrasonic files. Solvents (A and B Eugesolv, Septodont) were used to facilitate root canal filling removal if necessary. The smear layer was removed by EDTA (Chelasolv, Septodont) and the canals were dried with sterile paper points. Perforations were filled with Mineral Trioxide Aggregate (MTA ProRoot, Dentsply).

For teeth with preoperative apical periodontitis, the treatment was performed in one session if the chemomechanical preparation of the root canal system was complete, if the tooth was asymptomatic before the appointment, and if the canal was dry. If not, calcium hydroxide was introduced into the canals by hand file or using a Lentulo file.

Root canals were filled by vertical compaction using the Shilder technique [8] or only for downpacking and by means of a Gutta-condensor (Maillefer, cones of gutta-percha-Henry Schein Maxima, Pulp Canal Sealer-Kerr, Machtou pluggers Dentsply, Touch'n-Heat Sybron Endo).

Glass ionomer cement (Fuji IX Fast, GC corp.) was placed as temporary coronal seal and it was recommended to the patient to see his dentist to implement quickly coronal restoration. In some cases, treatment was protected by a temporary crown sealed by polycarboxylate cement (Durelon, 3 M ESPE).

All patients were treated under the same conditions (same dental office, same materials and instrumentation, and same operating protocols). The only variables were the operator and the patient.

### 2.3. Recall

Patient listing was made from postoperative written reports. The telephone numbers of patients were registered with the software Agenda Web (AP-HP). Patients were only contacted by telephone; a message was left on answering machine if no response. In case of missed call, we tried two attempts to contact the patient.

Recall was also standardized and made by two calibrated senior practitionners.

### 2.4. Radiographic Calibration

Two independent investigators studied all the anonymized X-Rays and were previously calibrated against a set of 100 reference teeth. Afterwards, they examined all the radiographs independently. A third observer, a highly experienced endodontist, was consulted for cases for which disagreement occurred.

### 2.5. Data

Clinical and radiographic pre-intra and immediate postoperative data were normally recorded in the patient medical record of each patient at the time of treatment by the operator (postgraduate practitioners supervised by the Professor director of the postgraduate cursus). Patients were then invited to a clinic/radiographic recall. Data previously recorded and subsequent data recorded at recall were pooled and evaluated.

**Figure 1:**
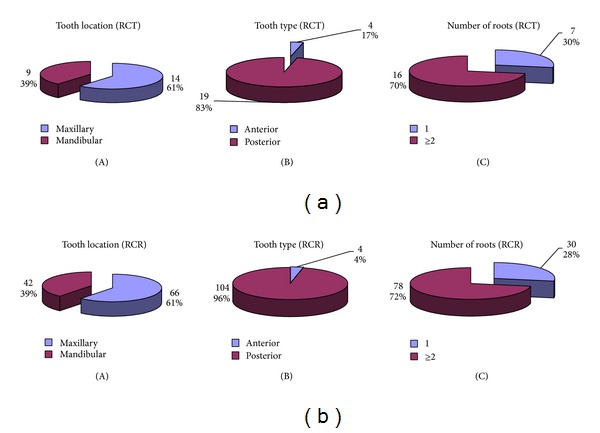
Tooth specifications. (a) For the root canal treatment (23 teeth): tooth location (A), tooth type (B), and number of roots (C). (b) For the root canal retreatment (108 teeth): tooth location (A), tooth type (B), and number of roots (C).

Demographic characteristics (age and gender) and tooth characteristics (tooth location, type of tooth, and number of roots) were recorded (Figure 1).

Preoperative conditions collected were clinical signs and symptoms, pulp vitality, presence and size of Apical Periodontitis (AP), root filling material, root filling length, root filling density, presence of a perforation and/or fractured instrument, and time elapsed since the initial endodontic treatment.

Intraoperative characteristics recorded were number of treatment sessions, root filling length, root filling density, presence of a perforation, and/or fractured instrument.

Postoperative characteristics were clinical signs and symptoms, presence and size of periapical radiolucency, type of coronal-root restoration, presence of post, restoration leakage, and fracture and were recorded the day of postoperative control. This was carried out between 1 and 4 years after endodontic treatment for each tooth treated. Clinical and radiographic evaluation was performed by two reviewers. The radiographs were scored according to the PAI system [9] and AP were classified as absent (PAI = 1), or present (PAI ≥ 2).

In multirooted teeth, the condition of the most severely affected root was considered (Table 1).

### 2.6. Outcome Assessment

Included patients were seen during a consultation in the Dental Clinic of Bretonneau Hospital by the examiner. During this consultation, clinical and radiographic examinations were performed.

The extracted teeth were excluded from the analysis of results, because reason for extraction could not be determined. We included in the study the remaining included teeth at the consultation day (whether received initial treatment or retreatment).

The clinical and radiographic criteria were used for the results.

**Figure 2:**
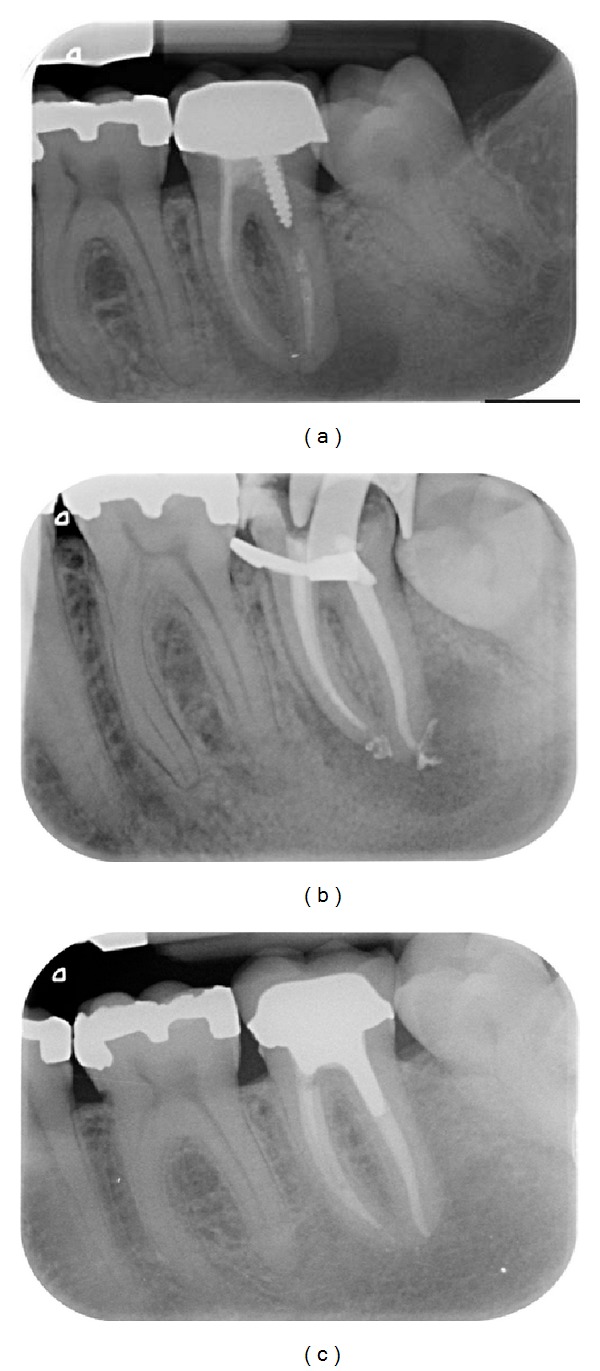
Case classified as healed. (a) Preoperative view of the 37, painful, with failure of an initial endodontic treatment performed more than one year ago. Note the apical radiolucency (PAI = 5). (b) Periapical X-ray control immediately after the endodontic retreatment. (c) Recall after 4 years. Note the disappearance of the apical radiolucency. This tooth is classified as “healed”: disappearance of pain and functional and asymptomatic tooth with complete healing of periapical lesion.

**Figure 3:**
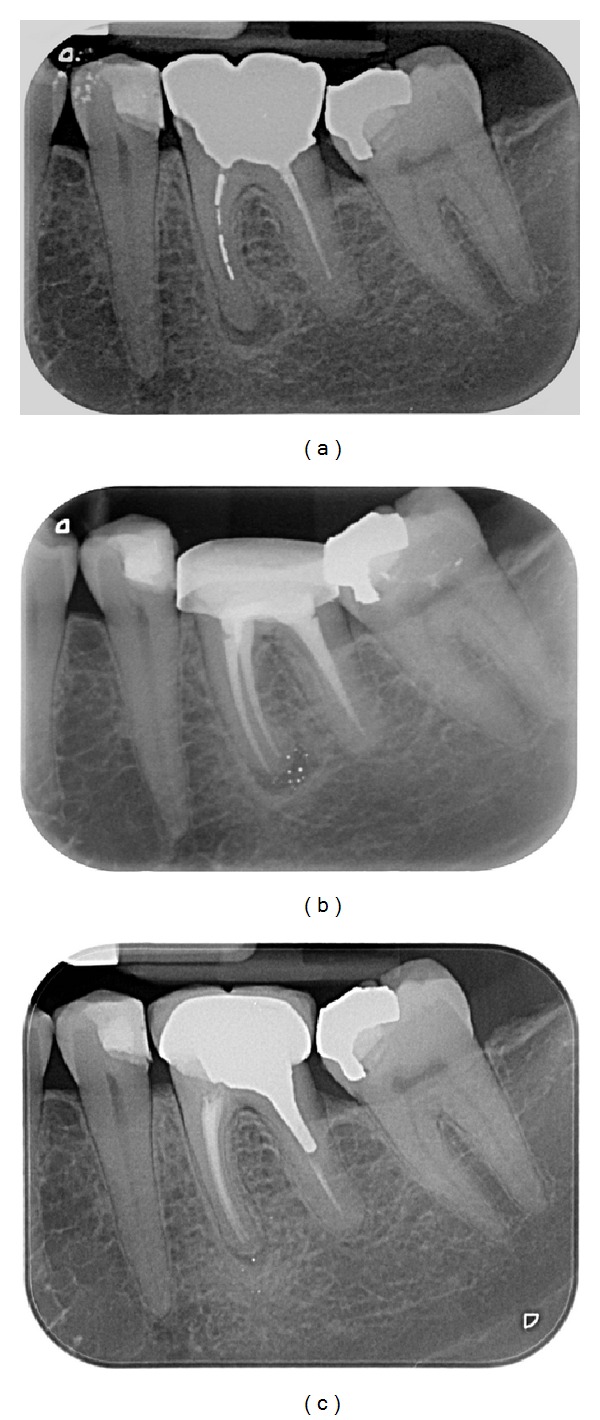
Case classified as healing. (a) Preoperative X-ray of the 36. Patient referred for orthograde retreatment of 36, asymptomatic, with an apical chronic periodontitis on the mesial root. Note the apical radiolucency (PAI = 3) and pieces of amalgam in the mesial root. The initial endodontic treatment was performed more than one year ago. (b) Periapical X-ray control immediately after the retreatment: amalgam grains are visible in the periapical region after realisation of the endodontic retreatment. (c) Recall after 4 years: this tooth is classified as “healed”: the amalgam grains seem to have disappeared but there is still a radiolucent area (PAI = 2).

**Figure 4:**
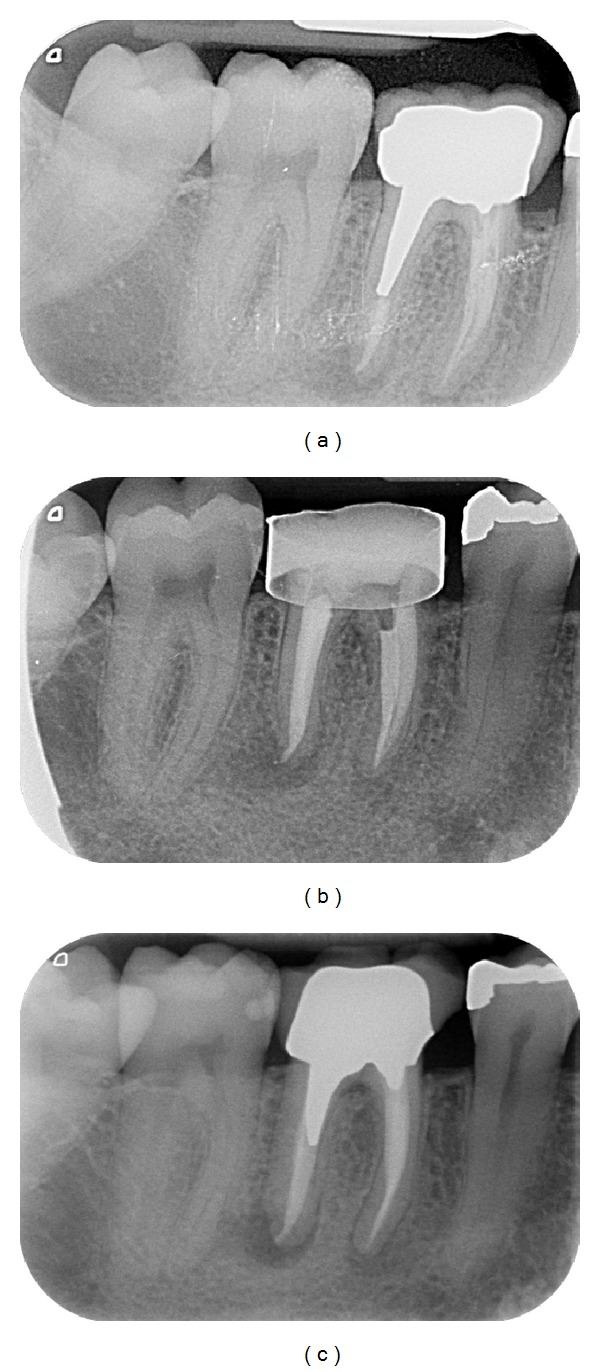
Case classified as failure. (a) Preoperative X-ray of the 46, painful, with an acute apical abcess. The previous endodontic treatment was performed 24 months ago. Note the apical radiolucency (PAI > 3). (b) Periapical X-ray control immediately after the retreatment. Note the copper ring used as preendodontic reconstitution. (c) Follow-up for 4 years: tooth was classified as “failure”: the apical radiolucency is always present and the tooth is symptomatic at the percussion test. The prosthodontics treatment and the coronal seal are questionable.

Endodontic success gathered “healed” teeth (absence of periapical radiolucency and no signs or symptoms) (Figure 2) and “healing” teeth (decrease of PAI score and absence of signs or symptoms) (Figure 3). Endodontic failures were characterized by “diseased teeth” and defined by presence of periapical radiolucency, signs, or symptoms (Figure 4). In these situations, a CBCT was required to clarify the failure and/or schedule an endodontic surgery (but the patient did not always carry out the examination).

Healing was considered “uncertain” if the patient described signs or symptoms as the tooth looked healed on the periapical X-ray. In these situations, a CBCT was prescribed.Then, after CBCT examination, the tooth was classified as follows:

- if there was no lesion on the treated tooth but another affected tooth could explain the symptoms, the treated tooth was considered as “cured”;
- if the CBCT revealed a lesion on the treated tooth not visible on intraoral radiograph, it was recorded as “not cured”.

### 2.7. Sample Size

All teeth evaluated were selected for statistical analysis. The sample size is the number of teeth and not the number of patients.

### 2.8. Statistical Analysis

We described the study population and we studied the bivariate associations between success and preoperative, intraoperative, and postoperative factors to identify potential outcome predictors. Fisher exact tests were conducted. All tests were interpreted at the 5% significance level. The number of failures was too small to have sufficient power to carry out multivariable analysis.* SAS software (9.2 version)* was used for analyses.

## 3. Results

Of the 296 patients (363 teeth treated), 148 patients were categorized as lost: one died; 25 changed phone number; 104 did not call back after the message left on their answering machine; 11 did not answer; and 7 patients' telephone numbers could not be retrieved from the database of the hospital.

148 patients (50% of the treated patients/of the eligible sample) were contacted: 7 patients had moved and could not come and 28 refused the follow-up visit (lack of availability, distance, and/or monitored regularly by a dentist). Ultimately, 113 patients were seen for visit. The recall rate is 38% (113 patients evaluated in 296 patients treated).

Sample of 113 patients and 139 teeth had been evaluated; 8 teeth extracted were excluded. Finally, 131 teeth were included in the study: 23 teeth had received an initial endodontic treatment and 108 teeth an orthograde retreatment.

The mean follow-up period was 35 months (±13.83).

The average number of treatments per patient was 1.88.

### 3.1. Distribution of the Sample

#### 3.1.1. Initial Endodontic Treatment

13 women (68%) and 6 men (32%), 47% of patients, were less than 45 years and 53% of patients were over 45 years.

Among the initial endodontic treatment, we treated mostly posterior teeth (83%), multirooted teeth (70%), and maxillary teeth (61%).

13% of patients described preoperative clinical signs or symptoms and preoperative apical periodontitis that was observed in 30% of teeth.

#### 3.1.2. Endodontic Retreatments

There were 51 women (59%) and 35 men (41%), 47% of patients, less than 45 years old and 53% of patients were over 45 years. The population who received retreatment was uniform regardless of age or gender. Among the 108 endodontic retreatments, we treated mostly posterior teeth (96%), teeth multirooted (72%), and maxillary teeth (61%).

39% of patients had preoperative clinical signs or symptoms. Preoperative apical periodontitis was observed on 66% of teeth.

Silver cones were observed for three teeth and gutta-percha for all other teeth. The majority of endodontic treatment was inadequate: 84% of short length and/or overfilling, 94% with voids.

### 3.2. Treatment Outcomes

The global (initial and retreatment) success rate was 92% (121 teeth). Initial endodontic treatments resulted in 100% success (23 teeth): for this reason, the statistical treatment of this study was conducted only on retreatments. Among the 108 retreated teeth, 80% (86 teeth) was healed and 11% (12 teeth) was healing. 9% (10 teeth) was diseased. Among the diseased teeth, two were fractured (Table 2).

### 3.3. Analysis of Preoperative Factors

Among the 42 teeth treated with preoperative signs and symptoms, the success rate was 83% (35 teeth), while for the 66 teeth without preoperative signs, there was 95% success rate. The success rate was statistically higher without preoperative signs and symptoms (*P* = 0,04), with an odd ratio of preoperative signs and symptoms on success, OR = 0.24, CI 95% [0.06; 0.98].

Of the 36 treated teeth without periapical radiolucency, 92% (33 teeth) was healed. Of the 72 teeth treated with preoperative AP, 90% (65 teeth) was healed or healing. But there was no statistically significant difference (Table 3).

#### 3.3.1. Evolution of the Size of AP

33 of the 36 teeth remained free of preoperative periapical radiolucency (94%) and 3 teeth developed apical periodontitis (6%).

Of the 24 teeth with preoperative PAI index = 2, 88% of periapical radiolucency disappeared (21 teeth), 8% of the lesions had the same PAI (2 teeth), and 4% of lesions increased in size (one tooth).

Of the 48 teeth with PAI index ≥3, the lesion size decreased for 92% (44 teeth) and remained stable for 8% (4 teeth).

#### 3.3.2. Root Filling Length and Voids

Of the 72 teeth with AP, a higher success rate was obtained when the preoperative root filling was short (92%) versus adequate (83%). It was not significantly different (*P* = 0.33). Of 108 retreated teeth, there was no significant association between preoperative root filling length and success.

Among 101 teeth with root filling voids, success rate was 94% and 57% if no void was recorded (7 teeth). The difference was statistically significant (*P* = 0,02).

#### 3.3.3. Treatment Complications

(i) Success rate was not different according to the preoperative presence of a broken instrument. Of 108 retreated teeth, 15 teeth had a preoperative broken instrument with a success rate of 87%. The 93 remaining teeth without broken instrument had a success rate of 91%.

(ii) Of the 108 retreated teeth, the two teeth with preoperative perforation healed.

(iii) In short, preoperative outcome predictors were signs and symptoms (absent, 95%: present 83%; *P* = 0,04) and root filling density (inadequate 93% versus 57%, *P* = 0,02).

The other preoperative factors did not seem to be outcome predictors in this study.

#### 3.3.4. Analysis of Intraoperative Factors

Of the 61 teeth treated in one session, the success rate was 89% while for the 47 teeth treated in two sessions, a success rate of 94% was obtained. There was no significant difference. However, the analysis of 72 teeth with preoperative periapical lesion gives a better result on the treated teeth in two sessions (97%) compared to those treated in one (86%).

Among the 89 teeth with preoperative short root canal filling, adequate length was found for 72 teeth (81%).

Of the 10 teeth treated with intraoperative complications (perforation or fractured instrument), there was no failure.

### 3.4. Analysis of the Factors Assessed at the Follow-Up Visit

No statistical difference was demonstrated relative to the presence of a post or to the quality of the coronary restoration.

### 3.5. Contribution of CBCT

91% of teeth (98 teeth) were asymptomatic the day of the visit; 9% of teeth (10 teeth) had clinical signs or symptoms. For the 10 symptomatic teeth, 4 teeth were considered not healed and 6 teeth as uncertain. A CBCT was prescribed to patients whose conclusion was uncertain. After viewing the 3D examination, two of them have been added to the successes and 4 to failures.

## 4. Discussion

In this retrospective study, the outcome of the endodontic treatment was assessed on both clinical and radiographic criteria, as recommended by the European Society of Endodontology [10]. The objective of an endodontic recall program is to discriminate between disease and health, cases in need of retreatment versus successful ones. In cases of lack of symptoms, survival analysis leads to success assessment even if a periapical lesion occurs. Alternately, the presence of an apical radiolucency does not signal a failure: the lesion is maybe decreasing or not mineralized. Informed consent must be given to the patient, and the need of postoperative recall sessions was highlighted.

### 4.1. Effectiveness

A new classification based on effectiveness/ineffectiveness was recently proposed [11]. Effective treatments (endodontic success) gather “healed” teeth (absence of AP and no signs or symptoms) and “healing” teeth (decrease of PAI score and absence of signs or symptoms). Ineffective treatments (endodontic failures) are characterized by “diseased teeth” and defined by presence of AP, signs, or symptoms.

In this study, effectiveness of initial treatment raised to 100% and to 91% for retreatment.

For initial treatment, success rate was 100% both for vital teeth (pulpitis) or nonvital teeth (necrosis and apical periodontitis). Previous study reported that outcome of endodontic treatment is different between vital and nonvital cases. Unfortunately this study cannot answer the question, due to a too small sample size that has statistical significance (only 23 teeth).

Healed teeth (80%) (see Figure 2) and healing teeth (11%) (see Figure 3) were considered as success. These data can be compared with those of the Toronto Study Phase 4 where 86% was considered as healed [2]. In another study, the main causes of failure when observed with an operative microscope during apical surgery were leaky canal or missing canal and underfilling and anatomical complexity [12].

**Figure 5:**
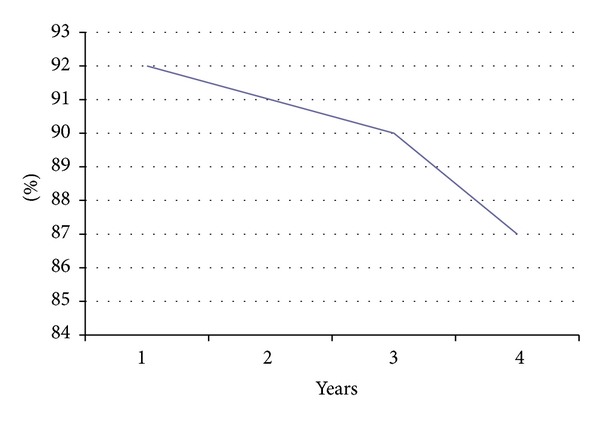
Diagram of success rate of the follow-up per year.

In this study, the success rate decreases by 5% over 4 years. The success rate is 92% 1 year after completion of endodontic treatment, 90% after 2 years, 91% after 3 years, and 87% after 4 years (Figure 5). This result can be explained either by the failure of endodontic treatment itself or as the result of restorative and prosthetic steps and subsequent complications. It emphasizes the need for controls on the long-term healing.

### 4.2. Radiographic Examination

Radiographic examinations allow the evaluation of the main success criterion and the presence or absence of a radiolucent image. 2D imaging underestimates the presence and the volume of AP and this is why a PAI score = 1 was chosen as an AP free indicator [13].

**Figure 6:**
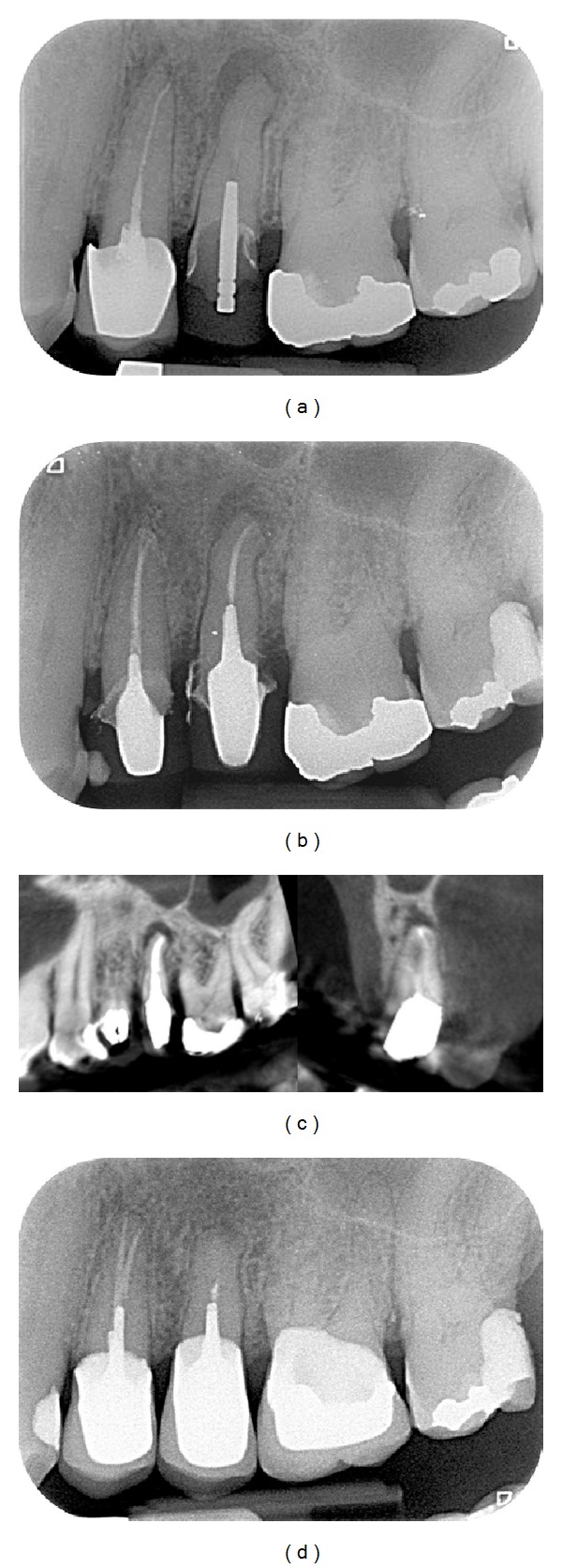
Contribution of the CBCT in the decision making process. (a) Periapical preoperative X-ray. Patient referred for the endodontic retreatment of 25. Note the periapical radiolucency on 25. (b) Control 12 months after the retreatment of 25. There are symptoms and persistent radiolucency on 25. The tooth was classified as “failure”. (c) CBCT coronal and sagittal slides of 25: a periapical surgery is indicated, (d) X-ray control 12 months after apical surgery and apical retrofilling with Biodentine (Septodont) which has the same radio-opacity as the root dentin.

Periapical lesions were scored with the PAI index [9]. The investigators can compare all the patient's radiographs. When the 2D X-ray cannot enable us to establish a diagnosis, a CBCT is prescribed to detect the presence of an apical radiolucency. On the 6 teeth classified as “uncertain”, the CBCT examination classified 4 teeth as failure. In this study the CBCT was not used as a method evaluating the results of treatment. The number of cases involved is small and therefore statistically not significant. However, the contribution of the CBCT is crucial to the accuracy of the diagnosis and the decision making process [13] (Figure 6). It poses, however, the problem of the radiographic evaluation of endodontic treatment and the need to move in the future towards 3D imaging to accurately monitor the result of treatment.

### 4.3. Apical Periodontitis (AP)

The presence or absence of an AP significantly affects the rate of success of endodontic treatment [14, 15]. In our study, we got 91% of success for teeth with AP and 92% of success for teeth without AP. These results may indicate that an experienced operator can obtain similar results, with or without AP. But the healing of an AP is an evolutionary process over several years which requires a period of observation of more than 3 years [6]. Periapical healing might take a long time to be achieved. If a lesion persists after 4 years, the root canal treatment was usually considered to be associated with posttreatment disease [10].

### 4.4. Quality of Initial Treatment

The length of preoperative root canal treatment was more than 2 mm from the apex on 82% of the retreated teeth (89 teeth), which is described as insufficient. A retrospective study in a German dental school has confirmed the negative impact of insufficient length on the success of endodontic treatment [16]. These results are consistent with other studies that conclude that nonsealed space is a haven for bacteria [17]. The presence of biofilms was also observed in these empty spaces and could be the cause of the persistence of AP [18]. Former root canal ledge is an additional obstacle to avoid during retreating. Theses ledges are generated by anatomical complexity and an inappropriate use of files, especially for undergraduate students [19]. 81% of the root canals with ledge could be prepared and sealed to the correct length in our study, which is in line with the 76% obtained in the Toronto Study phases 3 and 4 [20]. In our study, success rate reached 92% for teeth with AP and initial length of filling is too short. These good results are an encouragement to undertake root canal retreatment to shape and clean the apical portion not instrumented.

When the preoperative length was correct, the rate of success of our treatments was lower: 83%. It is probably the sign of a virulent infection less sensitive to retreatment procedures [17]. Another less common possibility would be the presence of an extraradicular infection, a true cyst or a reaction to a foreign body [21–24]. The planning of an endodontic surgery may be necessary in these situations.

### 4.5. Extrusions

For the 2 teeth with preoperative periapical overfilling of gutta-percha we obtained healing after retreatment. During the retreatment overfilling of gutta-percha that occurred in three cases, two teeth healed and one was healing. Among the teeth classified as failure, three are with extrusion of sealer. Among the teeth classified as healing four are with this kind of extrusion. The majority of teeth with sealer extrusion (21/28) are classified as healed. It's accepted that it is better to avoid overfilling but a controlled overfilling (“puff”) is not a predictor of failure.

### 4.6. Perforations

**Figure 7:**
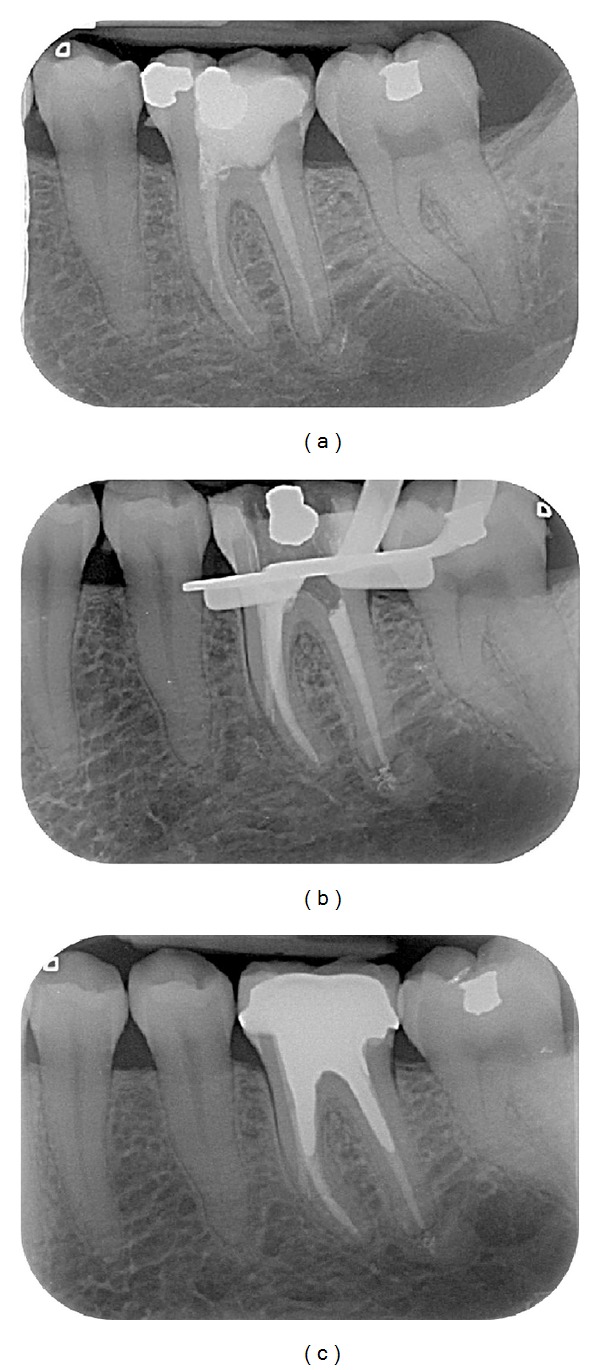
Successful endodontic retreatment of 36 symptomatic, with root perforation. (a) Preoperative X-ray: note the lateral periodontitis regarding the mesial perforation of the mesial root. (b) Peroperative X-ray: the perforation was filled with MTA and the root canal by vertical condensation of warm gutta-percha. (c) Follow-up for 1 year: tooth classified as “healed” (no symptoms, no radiolucency).

Perforations were long considered as major complications reducing the prognosis for survival of the tooth. Treated teeth from the Toronto Study have a better healing rate if the tooth is not perforated (86% versus 36%) [20]. These results are put in relation with the filling of these perforations material, namely, GIC cement. Since the use of MTA cement, the results are more predictable. In our study, all perforated teeth have recovered through the use of MTA cement and operative microscope. Thus, a perforated tooth may be kept on the arcade in so far as the perforation is accessible and its filling does not prevent access to the root canals (Figure 7).

### 4.7. Fractured Endodontic Instruments

**Figure 8:**
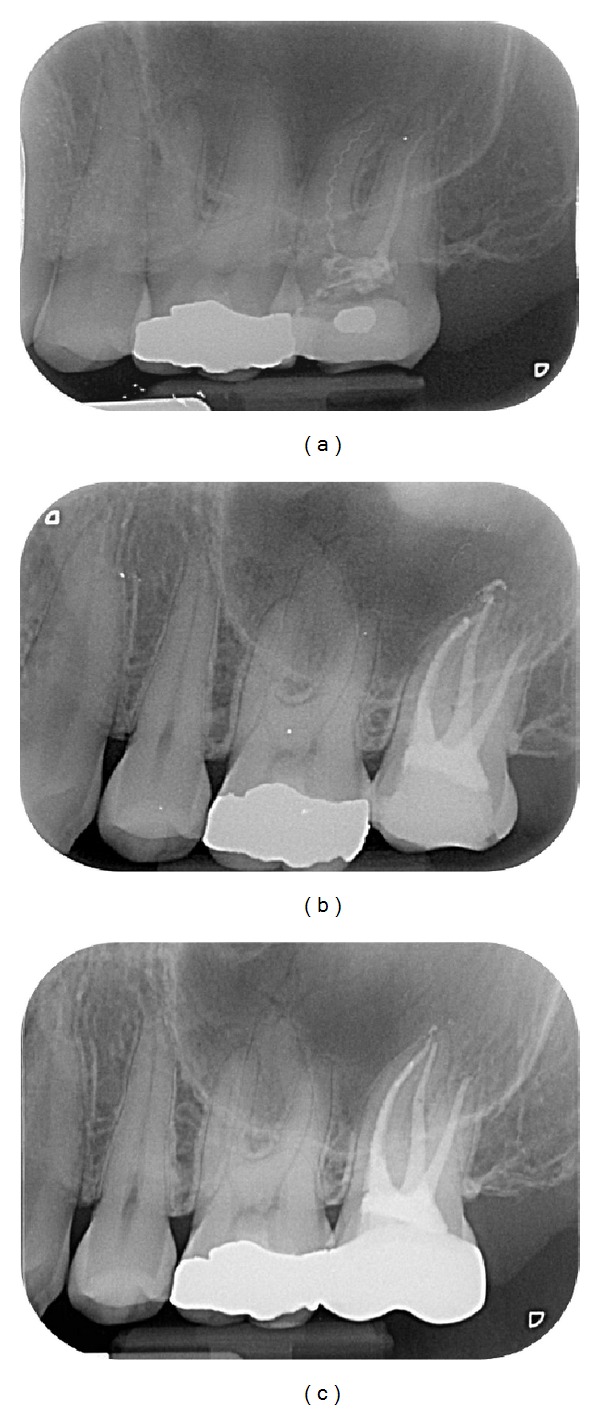
Successful endodontic retreatment of 27 symptomatic, with broken instrument. (a) Preoperative X-ray of a patient sent for orthograde retreatment of 27, painful, with broken instrument (Lentulo) in the mesio-buccal root. The initial treatment was recent. (b) Peroperative X-ray: the broken instrument has been removed and apical patency was found before the cleaning, shaping, and filling of the canal. (c) Recall X-Ray with follow-up for 4 years. Note that lateral radiolucency had disappeared. The tooth is classified as “healed”: absence of symptoms and periodontal healing.

The impact of a fractured instrument in a root canal must be assessed accurately. According to a recent systematic review [25], when endodontic treatment is performed to a high technical standard, the influence of a periapical lesion on the prognosis appears to be slight, but if the technical standard is compromised, the presence of a lesion can reduce the success rate considerably. In our study, the rate of success of retreatment with fractured instruments in a root canal is about 87%. Of the 15 teeth with preoperative broken instruments, two were removed and four were bypassed. Nine broken instruments were not removed and two failures occurred. During the retreatment five instruments were broken without any failure for these cases: the prognosis was favourable because fractures occurred after disinfection and apical patency (Figure 8).

Fractured file does significantly reduce healing of the lesion in the presence of AP. Therefore, although it is recommended that file removal should be attempted when possible, this does not appear to be evidence-based in the absence of apical disease [26].

### 4.8. Single or Multiple Treatment Sessions

For the “2-session” group, with a Calcium Hydroxide intersession dressing, a high rate of success was obtained (94%), while it was lower for treatment in one session (89%). In the presence of an AP, the probability of healing is here better for the multisession versus single session treatment (97% versus 86%) but the *P* value is 0.09 so the difference is not significant. A recent study [27] has demonstrated that the 2-visit protocol with an interappointment medication with calcium hydroxide resulted in improved microbiological status of the root canal system when compared with a single-visit protocol. On the basis of a systematic review, the healing rate of single- and multiple-visit root canal treatment is similar for infected teeth [28]. Therefore, no consensus can be established on this point.

### 4.9. Restoration Criteria

The quality of the coronary filling is an important factor in the prognosis of the treated teeth [29]. Its implementation must be done as soon as possible and it must guarantee the corono-radicular seal.

The presence of a post into the root canal is a frequently discussed factor. Our study demonstrates a rate of success of 89% for teeth with a post and 94% for those restored without post. The choice of a post should be considered a last-ditch attempt, and bacterial contamination should be avoided.

### 4.10. Health Condition

None of the referred patients presented any contraindication relative to endodontics. One patient had a specific disease (Hodgkin lymphoma). The tooth of this patient was extracted due to a root fracture.

## 5. Conclusion

Despite the limited sample size and the questionable recall rate, the results of this study confirm partially data from previous studies. It confirms the importance of identified predictors, including the initial symptoms and quality of initial treatment, as significant factors in the prognosis of the treatment. In the sample of patients involved, the presence of periapical radiolucency, the number of sessions, and the quality of the coronary restoration were not identified as statistically significant predictors.

The study investigates other factors such as the intraoperative incidents (broken instrument, perforations). However, for these factors due to the limited number of cases, the relative importance of each is not statistically significant. A larger sample size is needed to assess all outcome predictors of endodontics treatment more precisely.

Within the limits of this retrospective study, this work highlights the reliability of the initial endodontic treatment and the strong potential of endodontic retreatment when performed by trained and competent practitioners. The effectiveness of initial treatment is maximal and remains very high for retreatment.

## Figures and Tables

**Table 1:** Distribution of variables: preoperative, intraoperative, postoperative factors.

	Initial treatment		Retreatment	
	*N*	%	*N*	%
*Preoperative*				
Age				
≤45 years	9	47	40	47
>45 years	10	53	46	53
Gender				
Male	13	68	51	59
Female	6	32	35	41
Tooth type				
Anterior	4	17	4	4
Posterior	19	83	104	96
Tooth location				
Maxillary	14	61	66	61
Mandibular	9	39	42	39
No. of roots				
Single root	7	30	30	28
Multi-rooted	16	70	78	72
Signs and symptoms				
Absent	20	87	66	61
Present	3	13	42	39
Pulp response				
Yes	6	26	/	
No	2	9	/	
Unknown	14	61	/	
AP				
PAI = 1	15	65	36	33
PAI = 2	0	0	24	22
PAI ≥ 3	7	30	48	44
Root filling lenght				
Adequate	/	/	17	16
Short	/	/	89	82
Long	/	/	1	1
Mixed	/	/	1	1
Root filling density				
Adequate	/	/	7	6
Voids	/	/	101	94
Perforation				
Absent	/	/	106	98
Present	/	/	2	2
Broken instrument				
Absent	/	/	93	86
Present	/	/	15	14
Time since initial treatment				
≤1 year	/	/	21	19
>1 year	/	/	35	32
unknown	/	/	52	48
*Intraoperative*				
Treatment sessions				
Single-visit	19	83	61	56
Multi-visit	4	17	47	44
Root filling lenght				
Adequate	22	96	90	83
Short	1	4	14	13
Long	0	0	3	3
Mixed	0	0	1	1
Root filling density				
Adequate	23	100	104	96
Voids	0		4	4
Complications				
Perforation	1	4	5	5
Broken instrument	1	4	5	5
*Postoperative*				
Signs and symptoms				
Absent	22	96	98	91
Present	1	4	10	9
AP				
PAI = 1	23	100	89	82
PAI = 2	0	0	12	11
PAI ≥ 3	0	0	7	6
Coronal restoration				
Temporary filling	3	13	7	6
Permanent filling	20	87	101	94
Post				
Absent	16	70	36	33
Present	7	30	72	67
Restoration leakage				
Good	16	70	105	97
Poor	7	30	3	3
Fracture				
No	23	100	106	98
Yes	0	0	2	2

**Table 2:** Results.

	Initial treatment		Retreatment	
	*N*	%	*N*	%
Results*				
Healed	23	100	86	80
Healing	0	0	12	11
Diseased	0	0	10	9

*Including outcome after realisation of CBCT.

**Table 3:** Analysis of preoperative factors.

	Success						
	*N* ^ 1^	*n* ^ 2^	%	*P* ^ 3^	OR	CI_95%_	
Signs and symptoms							
Absent	66	63	95		1		
Present	42	35	83	0.04	0.24	0.06	0.98
AP							
PAI = 1	36	33	92		1		
PAI = 2	24	21	87		0.64	0.12	3.45
PAI > 3	48	44	92	0.83	1.00	0.21	4.78
Root filling length							
Adequate	17	14	82		1		
Short	91	84	92	0.19	2.57	0.59	11.14
Root filling density							
Adequate	7	4	57		1		
Voids	101	94	93	0.02	10.07	1.87	54.17
Complication*							
No	92	84	91		1		
Yes	16	14	87	0.64	0.67	0.13	3.14

^1^Number in each group.

^2^Number of success.

^3^ *P* of Fisher test.

*Broken instrument or perforation.
